# Supplementary material for: Comparative analysis of corrected tiger genome provides clues to its neuronal evolution
Source: Sci Rep. 2019 Dec 5;9:18459. doi: 10.1038/s41598-019-54838-z (PMC6895189; doi:10.1038/s41598-019-54838-z)
Supplement: Supplementary file 1 — Supplementary information [file 41598_2019_54838_MOESM1_ESM.docx]

**Supplementary Information**

**Title:** Comparative analysis of corrected tiger genome provides clues to its neuronal evolution

Authors: Parul Mittal^1^, Shubham K. Jaiswal^1^, Nagarjun Vijay^2^, Rituja Saxena^1^, Vineet K. Sharma^1^*

**Affiliation:**

^1^Metaomics and Systems Biology Lab, Department of Biological Sciences, Indian Institute of Science Education and Research Bhopal, Bhopal, India

^2^Computational Evolutionary Genomics Lab, Department of Biological Sciences, Indian Institute of Science Education and Research Bhopal, Bhopal, India

*Corresponding author email:

vineetks@iiserb.ac.in

**SUPPLEMENTARY FIGURES**

**
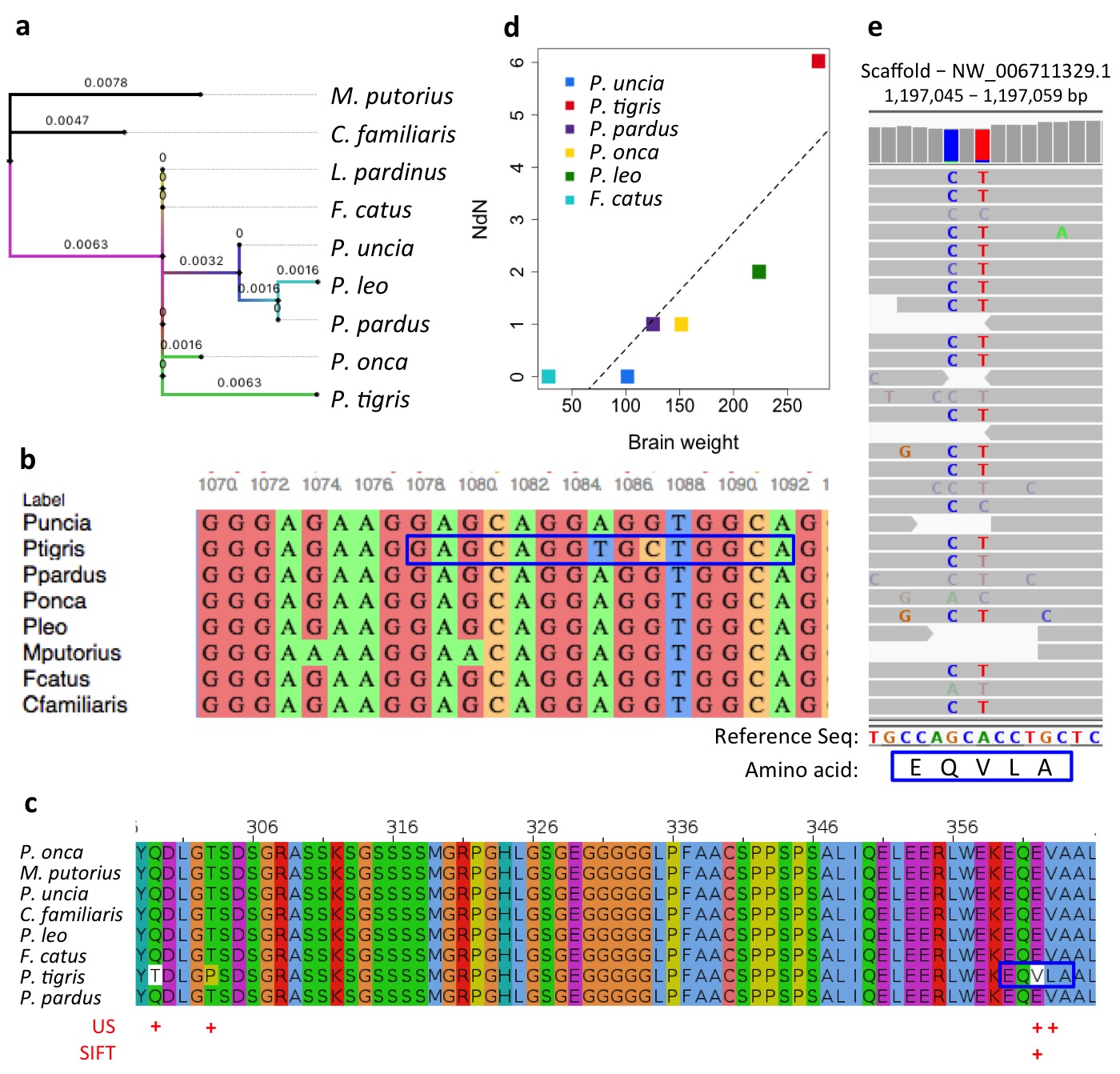
**

**Supplementary Figure S1. Multiple signs of adaptation in LZTS3 gene using the incorrect tiger geneset. a** higher branch length, **b** nucleotide alignment, **c** protein alignment showing unique substitutions (US) and substitution with functional impact (SIFT), **d** phylogenetically corrected correlation of LZTS3 gene with brain weight, **e** visualization of the site identified as having unique substitutions and substitution with functional impact in the gene in IGV. Two unique substitutions VL, with V as SIFT substitution in the blue box have been shown in the IGV along with its neighbouring sites. The gene exists on the (-) strand of the genome and its (+) strand is shown in **e**.

**
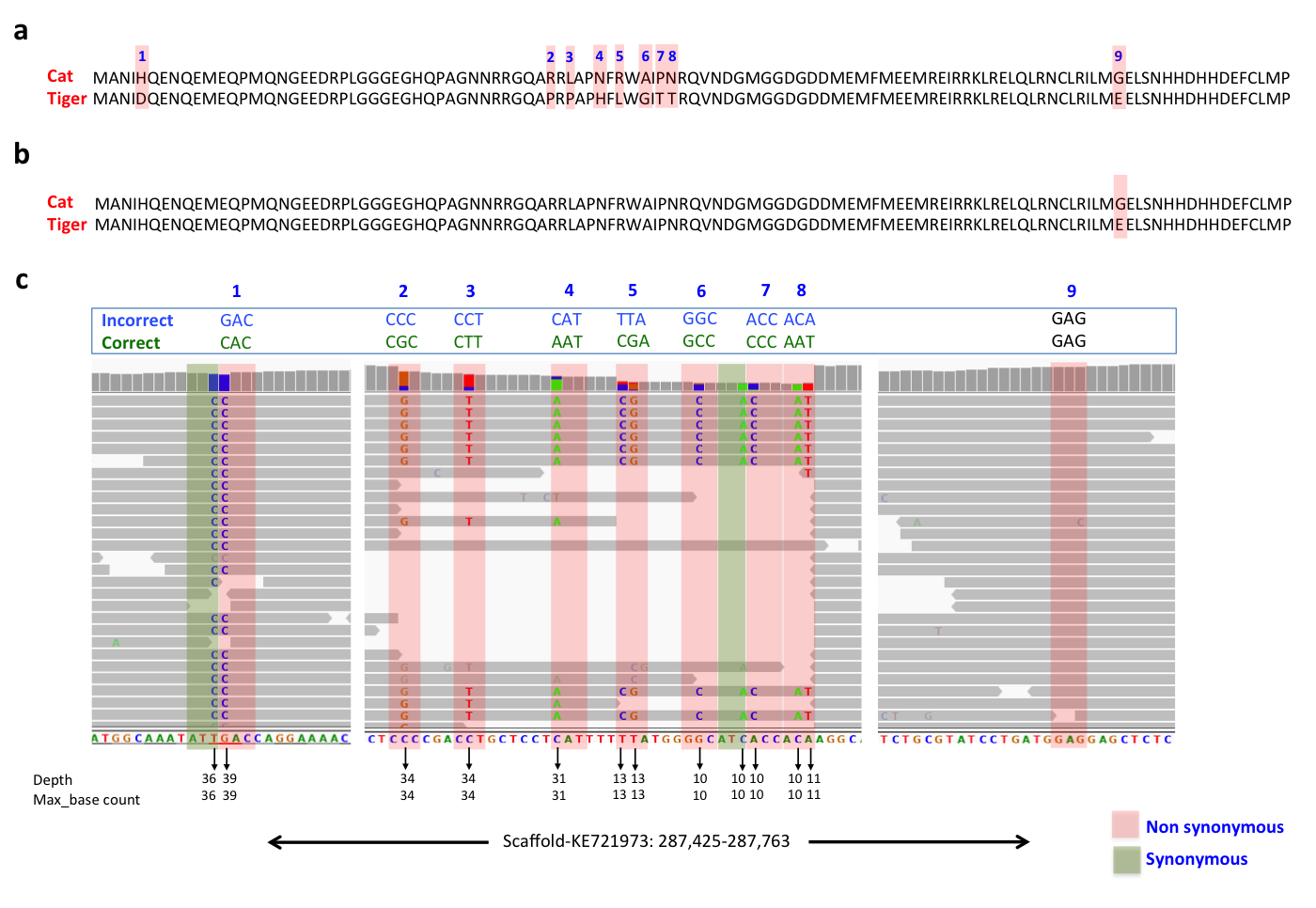
**

**Supplementary Figure S2: The BEX3 gene in tiger before and after correction. a** The unique amino acid substitutions in BEX3 gene in tiger in the incorrect version. **b** The unique amino acid substitutions in BEX3 gene in tiger in the corrected version. **c** Visualization of the nine substitutions (from incorrect version) in IGV. The sequence alignment has been taken from Ensembl release 94. The depth of each of the incorrect site and the corrected base count is shown at the bottom.

**
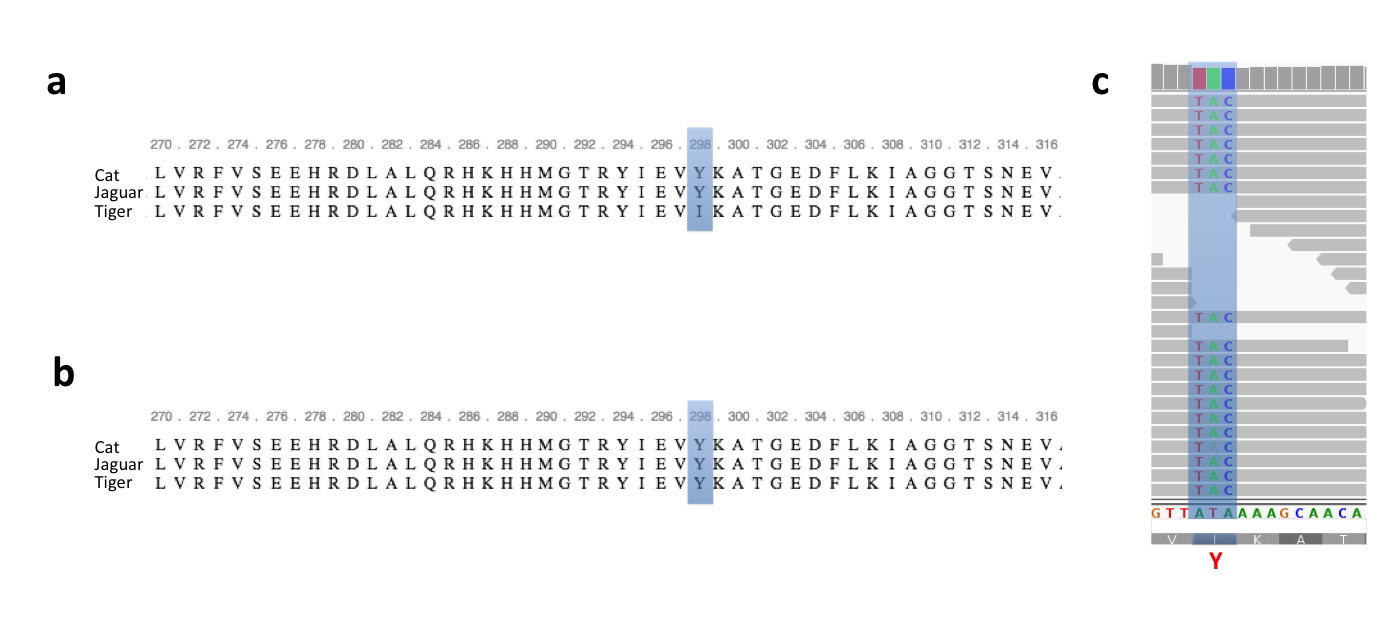
**

**Supplementary Figure S3. The divergence in ESRP1 gene involved in craniofacial robustness (Correction to Figueiró et al., 2017). a** The protein alignment for ESRP1 gene showing 298 position in felids using uncorrected tiger assembly. The highlighted 298 position in the alignment shows I298Y mutation in jaguar as identified by Figueiró et al., 2017. **b** The protein alignment for ESRP1 gene showing 298 position in felids using the corrected tiger assembly. **c** Reads alignment of tiger genome showing 298 position indicating that there is an error at this position in PanTig1.0 genome assembly version**.**

**
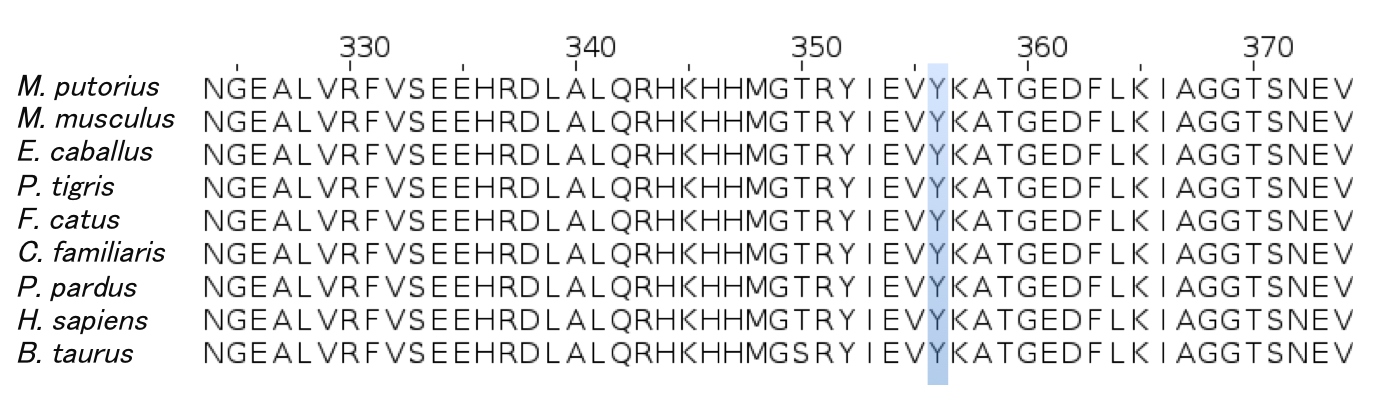
**

**Supplementary Figure S4. The protein alignment for ESRP1 gene showing 298 position in nine mammalian species (Correction to Figueiró et al., 2017).** An insertion of 58 amino acids at the beginning in *M. putorius* led to a shift in the alignment position to 356.

**
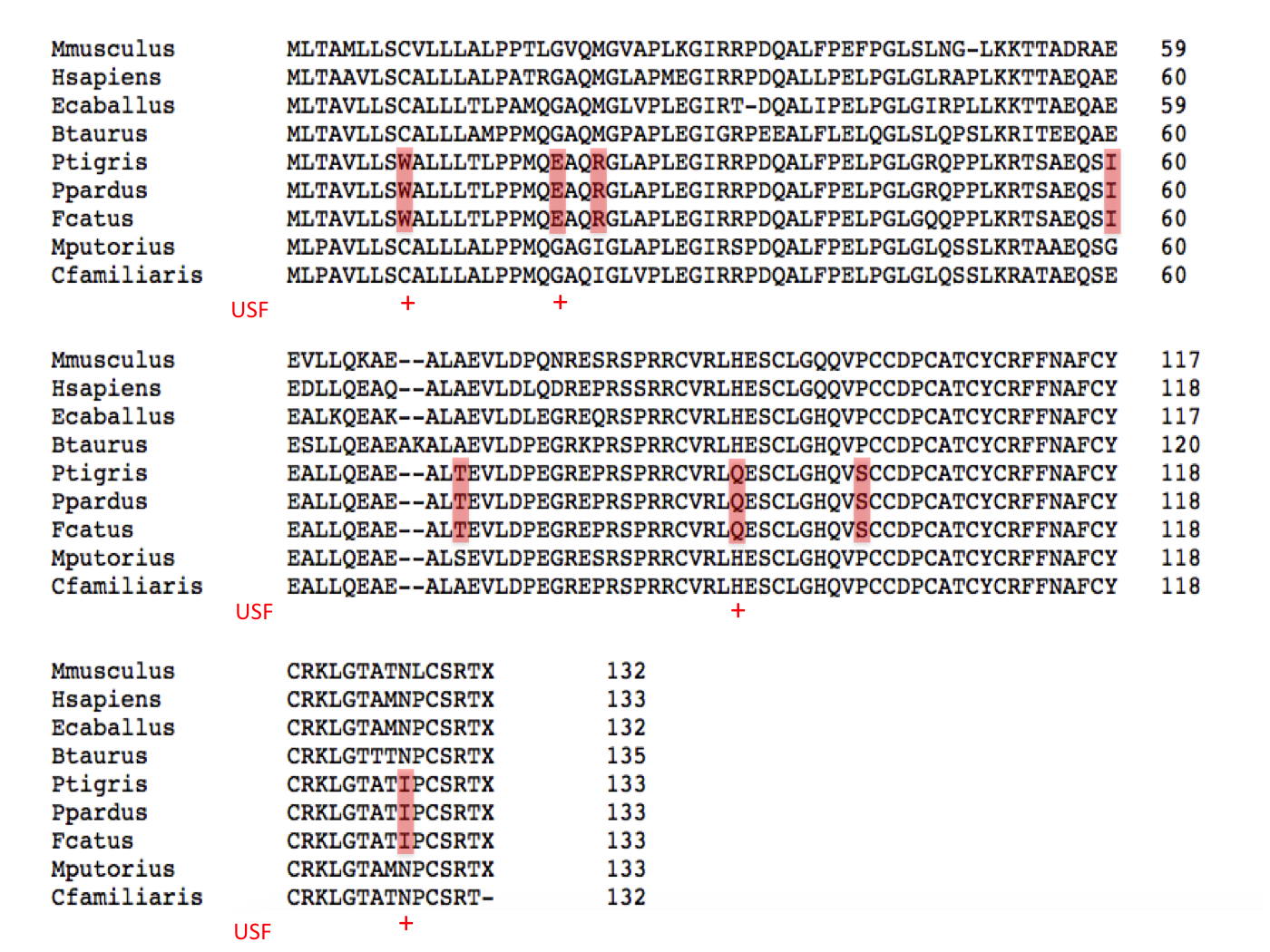
**

**Supplementary Figure S5. Alignment of AgRP gene orthologs from nine mammalian species showing felid specific amino acid substitutions.** The sites with unique substitution with functional impact (USF) in felids have been indicated with “+” sign. The gene in tiger (Ensembl gene id: ENSPTIG00000013563) is present at KE721923:10899097-10900020.

**SUPPLEMENTARY TABLES**

**Supplementary Table S1. List of 25 genes showing multiple signs of adaptation in tiger, as identified before correction in assembly/geneset. Errors were identified in all these genes when visualized using the IGV software.**

| **Gene Symbol** | **Description** | **Ensembl ID** | **Multiple Signs of Adaptation** | | | | **Erroneous positions** |
| --- | --- | --- | --- | --- | --- | --- | --- |
| PIK3CG | Phosphatidylinositol 4,5-bisphosphate 3-kinase catalytic subunit gamma isoform | ENSPTIT00000000115 | Higher dN/dS | Unique substitution | SIFT  (1 site) | - | 396;403;407;413;434;989 |
| HTR5A | 5-hydroxytryptamine receptor 5A | ENSPTIT00000018275 | Higher dN/dS | Unique substitution | - | - | 347 |
| BEX3 | Protein BEX3 | ENSPTIT00000009682 | Higher dN/dS | Unique substitution | SIFT  (1 site) | Branch length | 12;13;125;131;139;145;146;152;156;157;161;162 |
| IGSF9B | Protein turtle homolog B | ENSPTIT00000019071 | Higher dN/dS | Unique substitution | - | - | 3160;3162;3461 |
| LZTS3 | Leucine zipper putative tumor suppressor 3 | ENSPTIT00000003044 | Higher dN/dS | Unique substitution | SIFT  (1 site) | - | 225;366;627;805;810;843;983;985 |
| CHRDL1 | Chordin-like protein 1 | ENSPTIT00000009826 | Higher dN/dS | Unique substitution | SIFT  (1 site) | - | 515;521;536;541 |
| TENM1 | Teneurin-1 | ENSPTIT00000026194 | Higher dN/dS | Unique substitution | SIFT  (1 site) | - | 5845;5849 |
| FZD10 | Frizzled-10 | ENSPTIT00000000171 | Higher dN/dS | Unique substitution | SIFT  (1 site) | - | 1714;1715 |
| ERBB2 | Receptor tyrosine-protein kinase erbB-2 | ENSPTIT00000004011 | Higher dN/dS | Unique substitution | SIFT  (3 sites) | - | 1441;1442;1499;1500;1501;1502;1504;1505;1506;1507;2727;2728;3291;3611;3613 |
| PDZD11 | PDZ domain-containing protein 11 | ENSPTIT00000010393 | Higher dN/dS | Unique substitution | SIFT  (2 sites) | - | 407;413 |
| NHS | Nance-Horan syndrome protein | ENSPTIT00000022776 | Higher dN/dS | Unique substitution | SIFT  (3 sites) | - | 1248;1253;1301;2504;2505;2531;3831;3834;3880 |
| CPLX2 | Complexin-2 | ENSPTIT00000014485 | Higher dN/dS | Unique substitution | - | - | 142;173;177;179 |
| TGM6 | Protein-glutamine gamma-glutamyltransferase 6 | ENSPTIT00000001728 | - | Unique substitution | SIFT  (2 sites) | Branch length | 827;1077;1406;1409;1428 |
| CDK16 | Cyclin-dependent kinase 16 | ENSPTIT00000024023 | Higher dN/dS | Unique substitution | SIFT  (1 site) | Branch length | 486;1015;1024;1505;1540;1593;1594 |
| PEF1 | Peflin | ENSPTIT00000000994 | - | Unique substitution | SIFT  (1 site) | - | 136;138 |
| PLCB4 | 1-phosphatidylinositol 4,5-bisphosphate phosphodiesterase beta-4 | ENSPTIT00000001880 | - | Unique substitution | SIFT  (1 site) | - | 3376;3377 |
| ARID1A | AT-rich interactive domain-containing protein 1A | ENSPTIT00000019977 | Higher dN/dS | Unique substitution | SIFT  (1 site) | - | 348;351;358;370;3097;3112 |
| APBB1 | Amyloid beta A4 precursor protein-binding family B member 1 | ENSPTIT00000025188 | Higher dN/dS | Unique substitution | SIFT  (1 site) | - | 2110;2131 |
| RPS6KA6 | Ribosomal protein S6 kinase alpha-6 | ENSPTIT00000017915 | Higher dN/dS | Unique substitution | SIFT  (1 site) | - | 1219;1220;1222;1695;1707 |
| WFIKKN2 | WAP, Kazal, immunoglobulin, Kunitz and NTR domain-containing protein 2 | ENSPTIT00000023726 | Higher dN/dS | Unique substitution | - | - | 541;555;577;599;600;635;649 |
| CLIC1 | Chloride intracellular channel protein 1 | ENSPTIT00000016922 | Higher dN/dS | Unique substitution | SIFT  (2 sites) | Branch length | 196;197 |
| MCM6 | DNA replication licensing factor MCM6 | ENSPTIT00000029272 | Higher dN/dS | Unique substitution | SIFT  (2 sites) | - | 53;59 |
| GOLGA3 | Golgin subfamily A member 3 | ENSPTIT00000028800 | Higher dN/dS | Unique substitution | SIFT  (2 sites) | Branch length | 479 |
| TST | Thiosulfate sulfurtransferase | ENSPTIT00000030893 | Higher dN/dS | Unique substitution | SIFT  (1 site) | - | 53;55;57;59 |
| PUF60 | Poly(U)-binding-splicing factor PUF60 | ENSPTIT00000012363 | - | Unique substitution | SIFT  (1 site) | - | 1387;1389 |

**Supplementary Table S2: Summary of genome sequencing of Bengal tiger.**

| **Library Name** | **Library Preparation method** | **Insert Size** | **Sequencer** | **Read length** | **Raw reads** |
| --- | --- | --- | --- | --- | --- |
| TG-1 | Nextera XT | 650 bp | NextSeq 500 | 150*2 | 21,003,653 |
| TG-2 | Nextera XT | 650 bp | NextSeq 500 | 150*2 | 27,243,831 |
| TG-3 | Truseq PCR free | 550 bp | NextSeq 500 | 150*2 | 52,406,157 |
| CTG | Truseq PCR free | 350bp | HiSeq | 250*2 | 75,027,209 |

**Supplementary Table S3: Summary of transcriptome sequencing of Bengal tiger.**

|  | **Library preparation method** | **Sequencer** | **Raw reads** |
| --- | --- | --- | --- |
| Library 1 | TruSeq RNA Sample prep Kit | HiSeq 2500 | 15,823,343 |
| Library 2 | TruSeq RNA Sample prep Kit | HiSeq 2500 | 16,429,561 |

**Supplementary Table S4: Sequence statistics of data used for mapping against tiger and leopard genome assemblies**

|  | **Sequence data used in mapping** | | | **Reference assembly** | | |
| --- | --- | --- | --- | --- | --- | --- |
|  | **Sequence coverage** | **Insert sizes** | **Total data** | **Estimated genome size** | **Assembly size** | **Assembly version** |
| Amur Tiger | 83 x | 170, 500, 800 bp | 203 Gb | 2.44 Gb | 2.33 Gb | PanTig1.0 |
| Bengal Tiger | 27 x | 350, 550, 650 bp | 67 Gb |  |  |  |
| Leopard | 153 x | 170, 400, 500, 700 bp | 375 Gb | 2.45 Gb | 2.48 Gb | PanPar1.0 |

**Supplementary Table S5: Optimization of the parameters for SeqBug pipeline.** The table shows the similarity score between cat and tiger nucleotide sequences of 11 randomly selected sites in the tiger genome. The corresponding cat homologous region was selected and aligned with the corrected tiger genomic region for each iteration. The parameter used in the third iteration was selected based on its overall best performance nucleotide sequence.

| **Iterations** | **Criteria** | **Number of bases showing similarity with cat** |
| --- | --- | --- |
| 1 | Ref_base = 0 | 92453 |
| 2 | Ref_base ≤ Max_base/10 | 101903 |
| 3 | **Ref_base ≤** **Max_base/5** | **117592** |
| 4 | Ref_base ≤ Max_base/3.33 | 95556 |
| 5 | Ref_base ≤ Max_base/2.5 | 92458 |

**Supplementary Table S6: Global statistics of genomes used in the analysis**

|  | **Base pairs** | **N50 (scaffold)** | **N50 (contig)** | **Assembly version** | **Coding genes** |
| --- | --- | --- | --- | --- | --- |
| Tiger | 2,332,832,693 | 8,860,407 | 30,032 | PanTig1.0 | 17,544 |
| Leopard | 2,478,865,742 | 21,701,857 | 20,993 | PanPar1.0 | 19,688 |
| Cat | 2,491,009,630 | 83,967,707 | 41,915,695 | Felis_catus_9.0 | 19,446 |
| Dog | 2,392,715,236 | 45,876,610 | 267,478 | CanFam3.1 | 19,857 |
| Cow | 2,649,685,036 | 6,380,747 | 96,951 | UMD3.1 | 19,997 |
| Ferret | 2,277,906,570 | 9,335,154 | 44,823 | MusPutFur1.0 | 19,910 |
| Horse | 2,428,790,173 | 87,230,776 | 1,502,753 | EquCab2 | 20,449 |
| Human | 3,609,003,417 | 67,794,873 | 57,879,411 | GRCh38 | 20,454 |
| Mouse | 3,486,944,526 | 52,589,046 | 32,273,079 | GRCm38 | 22,480 |

**Supplementary Table S7: Adaptive evolution in felids. The top 20 GOs identified as enriched in positive selection and higher branch dN/dS (HBW) analyses in felids after correction.**

| **Positive selection** | | | **HBW** | | |
| --- | --- | --- | --- | --- | --- |
| **Description** | **O** | **p-value** | **Description** | **O** | **p-value** |
| stress-activated protein kinase signaling cascade | 30 | 0.0001 | sensory perception of mechanical stimulus | 21 | 0.0001 |
| regulation of protein serine/threonine kinase activity | 47 | 0.0001 | synapse organization | 23 | 0.0005 |
| regulation of gene expression, epigenetic | 25 | 0.0003 | establishment or maintenance of cell polarity | 18 | 0.0007 |
| positive regulation of kinase activity | 48 | 0.0004 | organelle localization | 39 | 0.0012 |
| sensory perception of mechanical stimulus | 21 | 0.0007 | muscle cell differentiation | 31 | 0.0019 |
| organ or tissue specific immune response | 5 | 0.0007 | vesicle-mediated transport in synapse | 14 | 0.0019 |
| neuron projection guidance | 24 | 0.0016 | multi-organism metabolic process | 15 | 0.0020 |
| tumor necrosis factor superfamily cytokine production | 13 | 0.0018 | morphogenesis of an epithelial sheet | 8 | 0.0025 |
| sensory organ development | 46 | 0.0025 | sensory organ development | 41 | 0.0026 |
| gene silencing | 22 | 0.0025 | regulation of binding | 26 | 0.0026 |
| axon development | 40 | 0.0030 | mesenchyme development | 22 | 0.0030 |
| cell-cell signaling by wnt | 40 | 0.0040 | stress-activated protein kinase signaling cascade | 23 | 0.0032 |
| maintenance of location | 26 | 0.0043 | neurotransmitter transport | 19 | 0.0034 |
| regulation of intracellular transport | 40 | 0.0062 | respiratory system development | 20 | 0.0037 |
| embryonic organ development | 36 | 0.0082 | response to inorganic substance | 36 | 0.0053 |
| regulation of binding | 27 | 0.0086 | regulation of neurotransmitter levels | 18 | 0.0070 |
| tube morphogenesis | 31 | 0.0096 | developmental growth involved in morphogenesis | 20 | 0.0074 |
| beta-catenin-TCF complex assembly | 6 | 0.0098 | urogenital system development | 27 | 0.0075 |
| inclusion body assembly | 4 | 0.0099 | connective tissue development | 21 | 0.0077 |
| syncytium formation | 8 | 0.0100 | trabecula morphogenesis | 8 | 0.0084 |

**Supplementary Table S8: Adaptive evolution in *Panthera*. The top 20 GOs identified as enriched in positive selection, unique substitution with functional impact (USF) and higher branch dN/dS (HBW) analyses in after correction.**

| **Positive selection** | | | **USF** | | | **HBW** | | |
| --- | --- | --- | --- | --- | --- | --- | --- | --- |
| **Description** | **O** | **p-value** | **Description** | **O** | **p-value** | **Description** | **O** | **p-value** |
| stress-activated protein kinase signaling cascade | 43 | 0.0000 | sperm motility | 12 | 0.0000 | tube morphogenesis | 39 | 0.0001 |
| regulation of chromatin organization | 25 | 0.0020 | cilium organization | 25 | 0.0017 | developmental growth involved in morphogenesis | 27 | 0.0004 |
| regulation of protein serine/threonine kinase activity | 62 | 0.0022 | ventricular system development | 5 | 0.0092 | cell-cell signaling by wnt | 45 | 0.0005 |
| regulation of gene expression, epigenetic | 32 | 0.0022 | regulation of response to wounding | 14 | 0.0114 | regulation of chromatin organization | 20 | 0.0005 |
| sensory organ development | 68 | 0.0022 | protein complex localization | 10 | 0.0132 | endocrine system development | 16 | 0.0006 |
| sensory perception of mechanical stimulus | 26 | 0.0068 | necrotic cell death | 6 | 0.0150 | sensory perception of mechanical stimulus | 22 | 0.0008 |
| pattern specification process | 47 | 0.0114 | neurotrophin signaling pathway | 5 | 0.0211 | Ras protein signal transduction | 35 | 0.0010 |
| dendritic spine organization | 10 | 0.0116 | lipid catabolic process | 22 | 0.0242 | stress-activated protein kinase signaling cascade | 28 | 0.0011 |
| embryonic organ development | 52 | 0.0132 | microtubule-based movement | 17 | 0.0277 | regulation of binding | 31 | 0.0012 |
| cell-cell signaling by wnt | 56 | 0.0134 | DNA repair | 34 | 0.0284 | synapse organization | 25 | 0.0013 |
| mesenchyme development | 32 | 0.0140 | cell-substrate adhesion | 24 | 0.0285 | connective tissue development | 27 | 0.0014 |
| protein maturation | 31 | 0.0169 | peptide cross-linking | 4 | 0.0292 | somite development | 13 | 0.0014 |
| neuron projection guidance | 30 | 0.0171 | sodium ion transport | 18 | 0.0294 | peptidyl-serine modification | 30 | 0.0017 |
| semaphorin-plexin signaling pathway | 8 | 0.0175 | neutral lipid metabolic process | 11 | 0.0310 | vesicle-mediated transport in synapse | 16 | 0.0018 |
| tube morphogenesis | 44 | 0.0187 | fatty acid metabolic process | 24 | 0.0327 | regulation of protein serine/threonine kinase activity | 44 | 0.0021 |
| somite development | 14 | 0.0194 | digestion | 11 | 0.0335 | gene silencing | 23 | 0.0021 |
| macromolecule deacylation | 14 | 0.0194 | skin development | 16 | 0.0350 | regulation of morphogenesis of an epithelium | 22 | 0.0023 |
| developmental growth involved in morphogenesis | 30 | 0.0203 | lipid homeostasis | 11 | 0.0361 | neural tube development | 19 | 0.0025 |
| skeletal system development | 57 | 0.0209 | response to nerve growth factor | 5 | 0.0400 | muscle cell differentiation | 35 | 0.0028 |
| insulin-like growth factor receptor signaling pathway | 8 | 0.0220 | interleukin-8 production | 6 | 0.0404 | respiratory system development | 23 | 0.0028 |

**Supplementary Table S9: The top 20 GO identified as enriched in genes of positively selected genes in tiger after correction.**

| **Geneset** | **Description** | **C** | **O** | **p-value** |
| --- | --- | --- | --- | --- |
| GO:0031098 | stress-activated protein kinase signaling cascade | 172 | 42 | 0.0001 |
| GO:0061053 | somite development | 58 | 16 | 0.0046 |
| GO:0071526 | semaphorin-plexin signaling pathway | 26 | 9 | 0.0064 |
| GO:0048568 | embryonic organ development | 286 | 55 | 0.0068 |
| GO:0097485 | neuron projection guidance | 151 | 32 | 0.0087 |
| GO:0071774 | response to fibroblast growth factor | 95 | 22 | 0.0098 |
| GO:0045995 | regulation of embryonic development | 84 | 20 | 0.0098 |
| GO:0050954 | sensory perception of mechanical stimulus | 118 | 26 | 0.0103 |
| GO:0198738 | cell-cell signaling by wnt | 312 | 58 | 0.0113 |
| GO:0040029 | regulation of gene expression, epigenetic | 143 | 30 | 0.0125 |
| GO:0007389 | pattern specification process | 252 | 48 | 0.0130 |
| GO:0097061 | dendritic spine organization | 34 | 10 | 0.0144 |
| GO:0030323 | respiratory tube development | 127 | 27 | 0.0145 |
| GO:0022604 | regulation of cell morphogenesis | 305 | 56 | 0.0160 |
| GO:0061564 | axon development | 307 | 56 | 0.0182 |
| GO:0006631 | fatty acid metabolic process | 226 | 43 | 0.0183 |
| GO:0030900 | forebrain development | 235 | 44 | 0.0223 |
| GO:0001101 | response to acid chemical | 223 | 42 | 0.0230 |
| GO:0007423 | sensory organ development | 362 | 64 | 0.0230 |
| GO:0071900 | regulation of protein serine/threonine kinase activity | 324 | 58 | 0.0234 |

**Supplementary Table S10: The top 20 GO identified as enriched in genes showing accelerated rate of evolution in tiger (as determined by higher branch dN/dS) after correction.**

| **Geneset** | **Description** | **C** | **O** | **p-value** |
| --- | --- | --- | --- | --- |
| GO:0061053 | somite development | 54 | 15 | 0.0001 |
| GO:0007507 | heart development | 332 | 51 | 0.0001 |
| GO:0007423 | sensory organ development | 339 | 51 | 0.0002 |
| GO:0050954 | sensory perception of mechanical stimulus | 108 | 22 | 0.0002 |
| GO:0031098 | stress-activated protein kinase signaling cascade | 158 | 28 | 0.0004 |
| GO:0060560 | developmental growth involved in morphogenesis | 145 | 26 | 0.0006 |
| GO:0097485 | neuron projection guidance | 142 | 25 | 0.0010 |
| GO:1902275 | regulation of chromatin organization | 97 | 19 | 0.0010 |
| GO:1903311 | regulation of mRNA metabolic process | 76 | 16 | 0.0011 |
| GO:0040029 | regulation of gene expression, epigenetic | 137 | 24 | 0.0013 |
| GO:0198738 | cell-cell signaling by wnt | 289 | 42 | 0.0015 |
| GO:0030900 | forebrain development | 221 | 34 | 0.0016 |
| GO:0035239 | tube morphogenesis | 230 | 35 | 0.0017 |
| GO:0016458 | gene silencing | 127 | 22 | 0.0023 |
| GO:0061564 | axon development | 287 | 41 | 0.0024 |
| GO:0051098 | regulation of binding | 186 | 29 | 0.0028 |
| GO:0071900 | regulation of protein serine/threonine kinase activity | 299 | 42 | 0.0029 |
| GO:0007265 | Ras protein signal transduction | 221 | 33 | 0.0030 |
| GO:0050803 | regulation of synapse structure or activity | 69 | 14 | 0.0033 |
| GO:1903008 | organelle disassembly | 69 | 14 | 0.0033 |

**Supplementary Table S11: GO enrichment of genes showing unique substitution with functional impact in tiger after correction.**

| **Geneset** | **Description** | **C** | **O** | **p-value** |
| --- | --- | --- | --- | --- |
| GO:0034067 | protein localization to Golgi apparatus | 20 | 8 | 0.0007 |
| GO:0050954 | sensory perception of mechanical stimulus | 119 | 22 | 0.0072 |
| GO:0071774 | response to fibroblast growth factor | 95 | 18 | 0.0112 |
| GO:0008202 | steroid metabolic process | 166 | 27 | 0.0174 |
| GO:0016358 | dendrite development | 137 | 23 | 0.0190 |
| GO:0051493 | regulation of cytoskeleton organization | 279 | 41 | 0.0216 |
| GO:0009410 | response to xenobiotic stimulus | 40 | 9 | 0.0230 |
| GO:0097061 | dendritic spine organization | 34 | 8 | 0.0243 |
| GO:0022604 | regulation of cell morphogenesis | 306 | 44 | 0.0250 |
| GO:0032886 | regulation of microtubule-based process | 96 | 17 | 0.0251 |
| GO:0044782 | cilium organization | 187 | 29 | 0.0256 |
| GO:0070849 | response to epidermal growth factor | 23 | 6 | 0.0304 |
| GO:0043062 | extracellular structure organization | 230 | 34 | 0.0317 |
| GO:0023019 | signal transduction involved in regulation of gene expression | 12 | 4 | 0.0320 |
| GO:0000226 | microtubule cytoskeleton organization | 295 | 42 | 0.0324 |
| GO:0050906 | detection of stimulus involved in sensory perception | 70 | 13 | 0.0330 |
| GO:0045730 | respiratory burst | 18 | 5 | 0.0365 |
| GO:0006631 | fatty acid metabolic process | 226 | 33 | 0.0395 |
| GO:0050951 | sensory perception of temperature stimulus | 13 | 4 | 0.0425 |
| GO:0051604 | protein maturation | 157 | 24 | 0.0458 |
| GO:0051235 | maintenance of location | 181 | 27 | 0.0466 |
| GO:0050953 | sensory perception of light stimulus | 142 | 22 | 0.0478 |

**Supplementary Table S12: The top 20 GO identified as enriched in genes showing higher nucleotide divergence in tiger (as determined by root-to-tip branch length method) after correction.**

| **Geneset** | **Description** | **C** | **O** | **p-value** |
| --- | --- | --- | --- | --- |
| GO:0048483 | autonomic nervous system development | 24 | 4 | 0.0004 |
| GO:0007423 | sensory organ development | 364 | 13 | 0.0025 |
| GO:0060485 | mesenchyme development | 164 | 8 | 0.0028 |
| GO:0061383 | trabecula morphogenesis | 41 | 4 | 0.0029 |
| GO:0021953 | central nervous system neuron differentiation | 108 | 6 | 0.0050 |
| GO:0051962 | positive regulation of nervous system development | 307 | 11 | 0.0053 |
| GO:0061564 | axon development | 309 | 11 | 0.0056 |
| GO:0071542 | dopaminergic neuron differentiation | 25 | 3 | 0.0056 |
| GO:1904888 | cranial skeletal system development | 49 | 4 | 0.0056 |
| GO:0097485 | neuron projection guidance | 152 | 7 | 0.0069 |
| GO:0001764 | neuron migration | 83 | 5 | 0.0073 |
| GO:0050954 | sensory perception of mechanical stimulus | 119 | 6 | 0.0079 |
| GO:0045787 | positive regulation of cell cycle | 201 | 8 | 0.0093 |
| GO:0021510 | spinal cord development | 59 | 4 | 0.0108 |
| GO:0061053 | somite development | 59 | 4 | 0.0108 |
| GO:0007389 | pattern specification process | 253 | 9 | 0.0119 |
| GO:0008637 | apoptotic mitochondrial changes | 64 | 4 | 0.0142 |
| GO:0051961 | negative regulation of nervous system development | 176 | 7 | 0.0147 |
| GO:0035265 | organ growth | 101 | 5 | 0.0162 |
| GO:0090287 | regulation of cellular response to growth factor stimulus | 143 | 6 | 0.0184 |

**Supplementary Table S13: eggNOG classification of MSA genes in tiger after correction.**

| **Category Code** | **eggNOG Category Description** | **Broad category** | **Number of genes** |
| --- | --- | --- | --- |
| [T] | Signal transduction mechanisms | Cellular processes and signaling | 219 |
| [K] | Transcription | Information storage and processing | 138 |
| [S] | Function unknown | Poorly characterized | 99 |
| [O] | Post-translational modification, protein turnover, and chaperones | Cellular processes and signaling | 76 |
| [U] | Intracellular trafficking, secretion, and vesicular transport | Cellular processes and signaling | 64 |
| [Z] | Cytoskeleton | Cellular processes and signaling | 64 |
| [W] | Extracellular structures | Cellular processes and signaling | 52 |
| [P] | Inorganic ion transport and metabolism | Metabolism | 51 |
| [B] | Chromatin structure and dynamics | Information storage and processing | 37 |
| [I] | Lipid transport and metabolism | Metabolism | 28 |
| [A] | RNA processing and modification | Information storage and processing | 27 |
| [G] | Carbohydrate transport and metabolism | Metabolism | 26 |
| [C] | Energy production and conversion | Metabolism | 16 |
| [D] | Cell cycle control, cell division, chromosome partitioning | Cellular processes and signaling | 12 |
| [E] | Amino acid transport and metabolism | Metabolism | 12 |
| [L] | Replication, recombination and repair | Information storage and processing | 11 |
| [F] | Nucleotide transport and metabolism | Metabolism | 10 |
| [J] | Translation, ribosomal structure and biogenesis | Information storage and processing | 10 |
| [V] | Defense mechanisms | Cellular processes and signaling | 8 |
| [Q] | Secondary metabolites biosynthesis, transport, and catabolism | Metabolism | 5 |
| [Y] | Nuclear structure | Cellular processes and signaling | 3 |
| [H] | Coenzyme transport and metabolism | Metabolism | 2 |
| [M] | Cell wall/membrane/envelope biogenesis | Cellular processes and signaling | 1 |
| [N] | Cell motility | Cellular processes and signaling | 1 |

**Supplementary Table S14: Pathway enrichment of MSA genes in tiger (XD-score). The top 20 pathway (based on XD-score) are shown after correction.**

| **Annotation**  (pathway/process) | **XD-score** | **Fisher q-value** | **Gene set size** | **Pathway size** | **Overlap size** |
| --- | --- | --- | --- | --- | --- |
| **Notch signaling pathway** | **1.4287** | 0.0091 | 846 | 47 | 11 |
| Biosynthesis of unsaturated fatty acids | 0.7579 | 0.7531 | 846 | 21 | 3 |
| Fc gamma R-mediated phagocytosis | 0.6602 | 0.2486 | 846 | 91 | 12 |
| Basal cell carcinoma | 0.6389 | 0.3004 | 846 | 55 | 8 |
| Adherens junction | 0.5988 | 0.3265 | 846 | 72 | 9 |
| Axon guidance | 0.5981 | 0.2221 | 846 | 128 | 16 |
| Other glycan degradation | 0.5972 | 0.9190 | 846 | 16 | 2 |
| Riboflavin metabolism | 0.5972 | 0.9190 | 846 | 16 | 2 |
| Base excision repair | 0.5631 | 0.7531 | 846 | 33 | 4 |
| Insulin signaling pathway | 0.5468 | 0.2367 | 846 | 134 | 16 |
| mTOR signaling pathway | 0.5310 | 0.5524 | 846 | 51 | 6 |
| Endometrial cancer | 0.5310 | 0.3476 | 846 | 52 | 7 |
| Long-term potentiation | 0.5310 | 0.4638 | 846 | 68 | 8 |
| Galactose metabolism | 0.5107 | 0.8794 | 846 | 26 | 3 |
| alpha-Linolenic acid metabolism | 0.4722 | 0.9741 | 846 | 18 | 2 |
| Melanogenesis | 0.4722 | 0.3380 | 846 | 99 | 11 |
| Phototransduction | 0.4722 | 0.8794 | 846 | 27 | 3 |
| Lysosome | 0.4472 | 0.3265 | 846 | 120 | 13 |
| B cell receptor signaling pathway | 0.4452 | 0.5147 | 846 | 74 | 8 |
| Glycosaminoglycan degradation | 0.4196 | 1.0000 | 846 | 19 | 2 |

**Supplementary Table S15: Tissue XD-score based on expression data of genes belonging to Notch pathway.**

| Tissue type | Tissue XD-score |  |  | Tissue type | Tissue XD-score |
| --- | --- | --- | --- | --- | --- |
| temporal lobe | 8.62 |  |  | amygdala | 1.78 |
| whole brain | 8.56 |  |  | parietal lobe | 1.69 |
| cerebellum peduncles | 8.21 |  |  | subthalamic nucleus | 1.65 |
| prostate | 8.08 |  |  | skeletal muscle | 1.64 |
| cerebellum | 4.44 |  |  | superior cervical ganglion | 1.49 |
| skin | 3.88 |  |  | trigeminal ganglion | 1.35 |
| placenta | 2.78 |  |  | medulla oblongata | 1.27 |
| globus pallidus | 2.42 |  |  | pons | 1.2 |
| atrioventricular node | 2.24 |  |  | cingulate cortex | 1.14 |
| liver | 2.22 |  |  | dorsal root ganglion | 1.11 |
| ciliary ganglion | 1.85 |  |  | prefrontal cortex | 0.79 |
| occipital lobe | 1.8 |  |  | testis | 0.47 |

**SUPPLEMENTARY TEXT**

**Supplementary Text S1: Need for error correction**

An orthologous gene set of nine species including tiger, constructed using Best reciprocal blast hit (BRBH) approach ^1^, was analysed to identify the genes showing branch-site positive selection, higher branch dN/dS, high nucleotide divergence (root-to-tip branch length), and unique amino acid substitutions with significant functional impact in tiger. A total of 25 genes showed the above mentioned multiple signs of adaptation in tiger (**Supplementary Table S4**). Among these, LZTS3 (Leucine Zipper Tumor Suppressor Family Member 3), a gene considered to play a crucial role in regulating the postsynaptic density of synapses, showed multiple signs of adaptation and a phylogenetically corrected correlation between NdN and brain weight (**Supplementary Figure S1**).

The identified unique amino acid substitutions in the 25 genes were mapped to the corresponding tiger genome assembly (http://tigergenome.org) and were found to be in accordance with the genome assembly. Further, these genes were manually evaluated for the existence of the identified substitutions by mapping genomic reads onto the genome assembly and visualized in Integrated Genome Viewer ^2^. In all these 25 genes, we observed that almost all of the substitutions were due to errors in the genome assembly. Furthermore, a similar validation was carried out using the latest version of the tiger assembly and gene set available at Ensembl release 94 (PanTig1.0). In this assembly version also, all the 25 genes showed similar erroneous substitutions. For example, two unique substitutions V and L at positions 362 and 363, respectively, in the LZTS3 gene were a result of errors in the assembly (**Supplementary Figure S1**). In this gene, the codon GAG was replaced with GTG and GTG was replaced with CTG in the (-) strand. Similarly, eight of nine unique substitutions in BEX3 gene were identified as errors in the assembly (**Supplementary Figure S2**). These observations suggests that the tiger genome assembly reported by Cho et al., 2013 ^3^ and at Ensembl release 94 comprised of several erroneous bases which were perhaps introduced by the *de novo* assembler or by the read correction tools. These genome assembly errors were primarily single nucleotide changes, of which many were present in the coding regions resulting in synonymous and non-synonymous changes. These errors led to misleading results in our evolutionary analysis performed using this tiger assembly and gene set.

*Methodology*

Nine species from the order Carnivora namely, *Mustela putorius*, *Canis familiaris*, *Felis catus*, *Lynx pardinus*, *Panthera tigris*, *Panthera uncia*, *Panthera pardus*, *Panthera leo* and *Panthera onca* were considered to identify evolutionary signatures in tiger. The gene sets for *M. putorius*, *C. familiaris* and *F. catus* were retrieved from Ensembl release 90. The gene orthologs for the five *Panthera* species were provided by Figueiró et al., 2017 ^4^. The gene set for lynx was provided by Abascal et al., 2016 ^5^. An orthologous gene set was constructed using BRBH approach ^1^ considering cat as a reference. The gene phylogeny of each ortholog was inferred from the species phylogeny. The protein alignment of each ortholog was carried out using SATé-II ^6^, which implemented PRANK for alignment, Muscle for merging the alignment and RAxML for tree estimation. The protein-based nucleotide alignment was carried out using TRANALIGN in EMBOSS package ^7^. The variation in ω ratio between lineages on individual genes was calculated using the free-ratio model in CodeML from the PAML software package (v4.9a) ^8^. The dN/dS values were calculated for the genes, which qualified likelihood ratio test using a conservative 5% false-discovery-rate criterion against the null model (One ratio). The genes showing higher dN/dS in tiger were identified using the branch model in PAML and the genes which qualified likelihood ratio test against null model (One ratio) were considered to show higher branch dN/dS. For investigation of positive selection, the branch-site model was used in PAML and the likelihood ratio was compared against the null model with 5% false discovery rate. Unique amino acid substitutions in tiger were identified using In-house scripts. Each of the identified substitutions were tested for their functional impact using Sorting Intolerant From Tolerant (SIFT) ^9^ using UniProt database as reference. The nucleotide divergence rate was calculated using root-to-tip branch length. The genes showing more than one sign of adaptation were considered for further analysis. The genes evolving with brain size were identified using phylogenetically corrected generalised least square fit in BayesTraits (v3.0.1) ^10^ (continuous regression) between selection pressure (NdN) and brain size.

**Supplementary Text S2**: **Impact of assembly errors in evolutionary analysis**

Since in our analysis, the incorrect assembly led to several misleading insights in the evolutionary context, we hypothesized that the results from previous studies, which used incorrect tiger assembly could also be due to assembly artefacts. Thus, we evaluated the results of such studies in the light of corrected tiger genome assembly.

Figueiró et al., 2017 ^4^ reported a gene “ESRP1” to be positively selected in jaguar affecting craniofacial robustness. They identified a unique substitution I298Y in jaguar with respect to other species, including tiger (incorrect assembly). We found that the “I” at this position in the tiger gene was an error and is “Y“ in the corrected assembly. Moreover, leopard, cat, dog and other mammals also contain “Y” at this position (**Supplementary Figure S3** and **S4**). Further, the study reports six genes from the Glypican pathway to be positively selected in tiger (using branch-site model). Among these six genes, we found that five genes namely, ARNT, GNA15, SIN3B, CA9 and TFDP1 had incorrect bases in their coding sequence, and were corrected in our study. These genes were not found to be positively selected after correction in our study. The authors further identified two genes, DOCK3 and COL4A5 to show the strongest signal of interspecies and intraspecies positive selection in jaguar after performing Bonferroni correction. We found that both these genes had incorrect bases in tiger. These genes were found to show interspecies introgression, which was confirmed by pair-wise divergence assessment in each *Panthera* species with cat in the windows of 100kb containing these genes. However, we found that a total of 226 bases were erroneous in the selected windows and the rate of error in these windows was 1.5 times greater than the average rate of the error in the complete tiger genome assembly. Thus, these results suggest that substantial corrections have been carried out in the studied region, which may significantly affect the interspecies adaptation and introgression analysis for these genes among *Panthera* and needs re-evaluation.

Dobrynin et al., 2015 ^11^ used the incorrect tiger genome assembly and gene set and calculated a distribution of genome-wide dN/dS values and compared it with *Acinonyx jubatus* (cheetah) genome. The results from this analysis suggest that cheetah distribution is significantly different from other species including tiger. However of these, 4,472 genes have changed in the corrected tiger assembly, which will change the distribution of dN/dS across the coding genome. However, we could not verify this due to the inaccessibility of the cheetah genome assembly and gene set.

Montague et al., 2014 ^12^ carried out evolutionary analysis to identify genes showing adaptive evolution in cat, felids and carnivores. The analysis was performed using the erroneous tiger genome assembly and identified 331 genes to be positively selected in felids, of which 294 genes could be compared with the present study based in their unambiguous assignment to Ensembl gene ID. Of these genes, 80 genes had erroneous substitutions in tiger. Our analysis identified only 44 genes to be positively selected (branch-site) or showed higher branch dN/dS (two branch model) in felids using branch-site model among the reported genes (Montague et al., 2014). The study also reports an enrichment of sensory perception related genes among PSGs in felids. Among the 14 genes reported (Montague et al., 2014) to show positive selection in felids with respect to sensory perception, only two genes (GUCA1A and GRIN2C) were found to be positively selected in felids after correction in tiger gene set.

Some of the finding in other studies such as in Mitra et al., 2019 ^13^ where the erroneous tiger assembly version PanTig1.0 was used for comparative analysis, could be biased and requires re-evaluation.

*Effect of assembly errors on divergence time*

A large number of erroneous bases were identified in this study, which can have significant impact on the divergence time calculation of tiger with its ancestors/other species. Using a mutation rate of 1.1 x 10^-9^ per base per year and genome size of 2.4 x 10^9^ ^3^, the mutation rate per year is 2.64, calculated as:

mutation rate per year  =  mutation rate per base per year   x genome size

A total of 982,606 bases were found erroneous in the tiger genome assembly. The upper limit by which these errors can affect the divergence time can be calculated as:

upper limit = erroneous bases x mutation rate per year

Using the above formula, it can affect the divergence time by 0.37 million years.

**Supplementary Text S3:** **Insights into felid, *Panthera* and tiger evolution**

*Insights into felid evolution*

Recent studies in felids have identified evolutionary signatures that are important for their unique sensory perception and hunting characteristics ^3,4,11,12,14^. However, these studies were performed using the previous gene set (from Cho et. al 2013) of tiger, which contained erroneous base substitutions that can potentially bias the findings. Thus, the usage of corrected tiger gene set in this study is expected to identify the signatures of adaptive evolution in felids. A total of 766 genes showed faster evolution and 906 genes showed positive selection in felid in comparison to the other mammals. These genes showed enrichment for biological functions such as sensory perception, neuronal functioning, cell signalling, development, and stress response (**Figure 2b**). The lists of statistically significant top-20 GO categories from the two analyses are provided in **Supplementary Table S7**. Several genes that previously showed adaptive evolution in felids could not be identified in this study, whereas many additional genes were found to have evolved in felid (**Supplementary Text S2**). However, previous studies on the evolution of felids have also reported positive selection and adaptive evolution in the genes involved in the sensory perception and neuronal functioning ^12^. This indicates that in terms of the broader biological processes, the results from the evolutionary analysis using the corrected genome assemblies corroborate with the previous study on felids ^12^.

*Insights into Panthera evolution*

The *Panthera* genus has shown a recent and rapid diversification, which now comprises of five species of modern big cats possessing several unique characteristics. To understand the genetic-basis of divergence within these species and as well as with respect to the other mammalian species, we performed the comparative evolutionary analysis of *Panthera* considering tiger and leopard, with seven other mammalian species. The analysis resulted in a total of 1,450 genes showing positive selection in *Panthera*, which were functionally enriched in sensory perception, regulation of protein serine/threonine kinase activity, gene expression regulation, stress response, and development. A total of 917 genes showed a faster rate of evolution (branch model), and were enriched in cell-cell signalling and early development functions. Further, 797 genes showed amino acid substitutions unique to *Panthera* with significant functional impact. These genes were enriched in the biological functions related to sperm motility, development, fatty acid metabolism, and DNA repair. The lists of statistically significant top-20 GO categories from the three analyses are provided in **Supplementary Table S8**. A previous study in *Panthera* had reported unique substitutions with functional impact in fatty acid metabolism and DNA repair categories ^3^, which were also observed in this study.

*Insights into tiger evolution*

A comprehensive analysis of the five types of evolutionary signals was performed using the gene orthologs identified from nine mammalian species to gain insights into the evolution of tiger. A total of 1,474 genes showed positive selection in tiger (branch-site model) and were enriched for functional categories such as early development, fatty acid metabolism and neuronal functioning (**Supplementary Table S9**). A total of 872 genes showed faster evolution in tiger (branch model) and were mainly enriched for functions related to organ development and sensory perception (**Supplementary Table S10**). A total of 1,158 genes showed unique substitutions with functional impact and were enriched for cell signalling, sensory perception, and cytoskeleton functions (**Supplementary Table S11**). A total of 151 genes showed high nucleotide divergence rate identified using root-to-tip branch length values in tiger. These genes were enriched for sensory perception, organ development, and neuronal related functions (**Supplementary Table S12**).

**Supplementary Text S4:** **Genome and transcriptome sequencing of the Bengal tiger**

Approximately 5-6 ml blood was drawn from the tail vein of a four years old male tiger at Van Vihar National Park, Bhopal, India and was collected in EDTA-coated vials. The fresh blood sample was immediately brought to the laboratory at 4 °C and genomic DNA was extracted using DNeasy Blood and Tissue Kit (Qiagen, USA) following the manufacturer’s protocol. Multiple shotgun genomic libraries were prepared using Illumina TruSeq DNA PCR-free library preparation kit and Nextera XT sample preparation kit (Illumina Inc., USA) as per the manufacturer’s instructions. The insert size for the TruSeq libraries was 350 and 550 bp, and the average insert size for Nextera XT libraries was ~650 bp. The insert size for both the libraries was assessed on 2100 Bioanalyzer using High Sensitivity DNA kit (Agilent, USA). The libraries were quantified using KAPA SYBR FAST qPCR Master mix with Illumina standards and primer premix (KAPA Biosystems, USA), and Qubit dsDNA HS kit on a Qubit 2.0 fluorometer (Life Technologies, USA) as per the recommended Illumina protocol. The normalised TruSeq 550 bp and Nextera XT libraries were loaded on Illumina NextSeq 500 platform using NextSeq 500/550 v2 sequencing reagent kit (Illumina Inc., USA) and 150 bp paired-end sequencing was performed. The TruSeq libraries of 350 bp were sequenced on Illumina HiSeq platform to generate 250 bp paired-end reads.

Total RNA extraction was carried out from the blood sample for transcriptomic analysis. The blood sample (~5 ml) was transferred into a 50 ml polypropylene conical centrifuge tube. The volume was brought up to 45 ml with 1x RBC Lysis Buffer (10x RBC Lysis Buffer: 89.9 g NH4Cl, 10.0 g KHCO3 and 2.0 ml 0.5 M EDTA dissolved in approximately 800 ml ddH2O and pH adjusted to 7.3) and incubated at room temperature for 10 minutes. The cells were pelleted at 600xg (~1,400 rpm) for 10 minutes in a room temperature centrifuge and the supernatant was discarded. The pellet was gently resuspended in 1 ml of RBC Lysis Buffer and transferred to a 1.5 ml microcentrifuge tube and incubated at room temperature for 5 minutes. The cells were pelleted for 2 minutes by centrifuging at room temperature at 3000 rpm. The supernatant was discarded, and the pellet was resuspended in 1 ml of sterile DPBS. The cells were again pelleted at room temperature at 3,000 rpm, and the supernatant was discarded. 1200 µl of TRIzol solution was added to each tube. 0.2 ml of chloroform was added, and the tube was vortexed for 15 seconds. The sample was then centrifuged at 13,000 rpm for 10 minutes at 4°C. The upper phase was removed and transferred to a clean microcentrifuge tube. To the remaining upper phase, an equal volume of cold isopropanol was added, and inverted to mix. The sample was placed in a -20°C freezer to precipitate. Sample was then centrifuged at 13,000 rpm for 10 minutes at 4°C. The supernatant was carefully discarded, and the pellet was rinsed with 0.5 ml of ice-cold 75% ethanol. The sample was centrifuged at 13,000 rpm for 10 minutes at 4°C. The supernatant was discarded, and the pellet was allowed to dry for 5 to 10 minutes to remove any remaining ethanol. The RNA pellet was dissolved by adding 20 µl of RNAse-free water. The transcriptomic libraries were prepared from the total RNA using the SMARTer universal low input RNA kit and TruSeq RNA sample prep kit v2 using the manufacturer’s instructions, and 100 bp paired end sequencing was performed on the Illumina HiSeq platform.

**References**

1 Wall, D., Fraser, H. & Hirsh, A. Detecting putative orthologs. *Bioinformatics* **19**, 1710-1711 (2003).

2 Thorvaldsdottir, H., Robinson, J. T. & Mesirov, J. P. Integrative Genomics Viewer (IGV): high-performance genomics data visualization and exploration. *Brief Bioinform* **14**, 178-192, doi:10.1093/bib/bbs017 (2013).

3 Cho, Y. S. *et al.* The tiger genome and comparative analysis with lion and snow leopard genomes. *Nat Commun* **4**, 2433, doi:10.1038/ncomms3433 (2013).

4 Figueiro, H. V. *et al.* Genome-wide signatures of complex introgression and adaptive evolution in the big cats. *Sci Adv* **3**, e1700299, doi:10.1126/sciadv.1700299 (2017).

5 Abascal, F. *et al.* Extreme genomic erosion after recurrent demographic bottlenecks in the highly endangered Iberian lynx. *Genome Biol* **17**, 251, doi:10.1186/s13059-016-1090-1 (2016).

6 Liu, K. *et al.* SATe-II: very fast and accurate simultaneous estimation of multiple sequence alignments and phylogenetic trees. *Systematic biology* **61**, 90-106 (2011).

7 Rice, P., Longden, I. & Bleasby, A. EMBOSS: the European molecular biology open software suite. *Trends in genetics* **16**, 276-277 (2000).

8 Yang, Z. PAML 4: phylogenetic analysis by maximum likelihood. *Molecular biology and evolution* **24**, 1586-1591 (2007).

9 Ng, P. C. & Henikoff, S. SIFT: Predicting amino acid changes that affect protein function. *Nucleic Acids Res* **31**, 3812-3814 (2003).

10 Pagel, M. & Meade, A. BayesTraits. *Computer program and documentation available at http://www. evolution. rdg. ac. uk/BayesTraits. html*, 1216-1223 (2007).

11 Dobrynin, P. *et al.* Genomic legacy of the African cheetah, Acinonyx jubatus. *Genome Biol* **16**, 277, doi:10.1186/s13059-015-0837-4 (2015).

12 Montague, M. J. *et al.* Comparative analysis of the domestic cat genome reveals genetic signatures underlying feline biology and domestication. *Proc Natl Acad Sci U S A* **111**, 17230-17235, doi:10.1073/pnas.1410083111 (2014).

13 Mitra, S. *et al.* De novo assembly and annotation of Asiatic lion (Panthera leo persica) genome. *bioRxiv*, 549790, doi:10.1101/549790 (2019).

14 Kim, S. *et al.* Comparison of carnivore, omnivore, and herbivore mammalian genomes with a new leopard assembly. *Genome Biol* **17**, 211, doi:10.1186/s13059-016-1071-4 (2016).

15 Sternson, S. M. Hypothalamic survival circuits: blueprints for purposive behaviors. *Neuron* **77**, 810-824 (2013).

16 Sternson, S. M. & Eiselt, A.-K. Three pillars for the neural control of appetite. *Annual review of physiology* **79**, 401-423 (2017).

17 Ilnytska, O. & Argyropoulos, G. The role of the Agouti-Related Protein in energy balance regulation. *Cell Mol Life Sci* **65**, 2721-2731, doi:10.1007/s00018-008-8104-4 (2008).

18 Vink, T. *et al.* Association between an agouti-related protein gene polymorphism and anorexia nervosa. *Mol Psychiatry* **6**, 325-328, doi:10.1038/sj.mp.4000854 (2001).

19 Marks, D. L. *et al.* Ala67Thr polymorphism in the Agouti-related peptide gene is associated with inherited leanness in humans. *Am J Med Genet A* **126A**, 267-271, doi:10.1002/ajmg.a.20600 (2004).

20 Van Valkenburgh, B. & Wayne, R. K. Carnivores. *Curr Biol* **20**, R915-919, doi:10.1016/j.cub.2010.09.013 (2010).

21 Sunquist, M. & Sunquist, F. *Wild cats of the world*. (University of chicago press, 2017).
